# A methodology for stochastic analysis of share prices as Markov chains with finite states

**DOI:** 10.1186/2193-1801-3-657

**Published:** 2014-11-06

**Authors:** Felix Okoe Mettle, Enoch Nii Boi Quaye, Ravenhill Adjetey Laryea

**Affiliations:** Department of Statistics, University of Ghana, Accra, Ghana; Department of Banking and Finance, University of Professional Studies, Accra, Ghana

**Keywords:** Markov process, Transition probability matrix, Limiting distribution, Expected mean return time, Markov chain

## Abstract

Price volatilities make stock investments risky, leaving investors in critical position when uncertain decision is made. To improve investor evaluation confidence on exchange markets, while not using time series methodology, we specify equity price change as a stochastic process assumed to possess Markov dependency with respective state transition probabilities matrices following the identified state pace (i.e. decrease, stable or increase). We established that identified states communicate, and that the chains are aperiodic and ergodic thus possessing limiting distributions. We developed a methodology for determining expected mean return time for stock price increases and also establish criteria for improving investment decision based on highest transition probabilities, lowest mean return time and highest limiting distributions. We further developed an R algorithm for running the methodology introduced. The established methodology is applied to selected equities from Ghana Stock Exchange weekly trading data.

**Electronic supplementary material:**

The online version of this article (doi:10.1186/2193-1801-3-657) contains supplementary material, which is available to authorized users.

## Background

Stock market performance and operation has gained recognition as a significantly viable investment field within financial markets. We most likely find investors seeking to know the background and historical behavior of listed equities to assist investment decision making. Although stock trading is noted for its likelihood of yielding high returns, earnings of market players in part depend on the degree of equity price fluctuations and other market interactions. This makes earnings very volatile, being associated with very high risks and sometimes significant losses.

In stochastic analysis, the Markov chain specifies a system of transitions of an entity from one state to another. Identifying the transition as a random process, the Markov dependency theory emphasizes "memoryless property" i.e. the future state (next step or position) of any process strictly depends on its current state but not its past sequence of experiences noticed over time. Aguilera et al. ( 1999) noted that daily stock price records do not conform to usual requirements of constant variance assumption in conventional statistical time series. It is indeed noticeable that there may be unusual volatilities, which are unaccounted for due to the assumption of stationary variance in stock prices given past trends. To surmount this problem, models classes specified under the Autoregressive Conditional Heteroskedastic (ARCH) and its Generalized forms (GARCH) make provisions for smoothing unusual volatilities.

Against the characteristics of price fluctuations and randomness which challenges application of some statistical time series models to stock price forecasting, it is explicit that stock price changes over time can be viewed as a stochastic process. Aguilera et al. ( 1999) and Hassan and Nath ( 2005) respectively employed Functional Principal Component Analysis (FPCA) and Hidden Markov Model (HMM) to forecast stock price trend based on non-stationary nature of the stochastic processes which generate the same financial prices. Zhang and Zhang ( 2009) also developed a stochastic stock price forecasting model using Markov chains.

Varied studies (Xi et al. 2012; Bulla et al. 2010; Ammann and Verhofen 2006; and Duffie and Singleton 1993) have researched into the application of stochastic probability to portfolio allocation. Building on existing literature, we assume that stock price fluctuations exhibit Markov’s dependency and time-homogeneity and we specify a three state Markov process (i.e. price decrease, no change and price increase) and advance the methodology for determining the mean return time for equity price increases and their respective limiting distributions using the generated state-transition matrices. We further replicate the case for a two-state space i.e. decrease in price and increase in price. Based on the methodology, we hypothesize that;

Equity with the highest state transition probability and least mean return time will remain the best choice for an investor.

We explore model performance using weekly historical data from the Ghana Stock Exchange (GSE); we set up the respective transition probability matrix for selected stocks to test the model efficiency and use.

## Review of theoretical framework

### Definition of the Markov process

The stochastic process {*X* (*t*), *tϵT*} is said to exhibit Markov dependence if for a finite (or countable infinite) set of points (*t*_0_, *t*_1_, … , *t*_*n*_, *t*), *t*_0_ < *t*_1_ < *t*_2_ < … < *t*_*n*_ < *t* where *t*, *t*_*r*_*ϵT* (*r* = 0, 1, 2, …, *n*).
1

From the property given by equation (1), the following relation suffices
2

where *t*_*n*_ < *τ* < *t* and *S* is the state space of the process {*X* (*t*)}.

When the stochastic process has discrete state and parameter space, (2) takes the following form: for *n* > *n*_1_ > *n*_2_ > … > *n*_*k*_ and *n*, *n*_*r*_*ϵT* (*r* = 1, 2, …, *k*)
3

A stochastic process with discrete state and parameter spaces which exhibits Markov dependency as in (3) is known as a Markov Process.

From the Markov property, for *n*_*k*_ < *r* < *n* we get
4

equations (2) and (4) are known as the Chapman-Kolmogorov equations for the process.

### n-step transition probability matrix and n-step transition probabilities

If *P* is the transition probability matrix of a Markov chain {*X*_*n*_, *n* = 0, 1, 2, …} with state space *S*, then the elements of *P*^*n*^ (*P raised to the power n*),  are the n-step transition probabilities where *P*_*ij*_^(*n*)^ is the probability that the process will be in state *j* at the *n*^*th*^ step starting from state *i*.

The above statement can clearly be shown from the Chapman-Kolmogorov equation (4) as follows; for a given *r* and *s*, write


Set *r* = 1, *s* = 1 in the above equation to get


Clearly, *P*_*ij*_^(2)^ is the (*i*, *j*)*th* element for the matrix product *P* × *P* = *P*^2^. Now suppose *P*_*ij*_^(*r*)^ (*r* = 3, 4, …, *n*) is the (*i*, *j*)^*th*^ of *P*^*r*^ then by the Kolmogorov equation, the


which again can be seen as the (*i*, *j*)^*th*^ element of the matrix product *P*^*r*^*P* = *P*^*r*+1^. Hence by induction, *P*_*ij*_^(*n*)^ is the (*i*, *j*)^*th*^ element of *P*^*n*^*n* = 2, 3, ….

To specify the model, the underlying assumption is stated about the identified n-step transition probability (stating without proof).

The transition probability matrix is accessible with existing state communication. Further, there exists recurrence and transience of states. States are also assumed to be irreducible and belong to one class with the same period which we take on the value 1. Thus the states are aperiodic.

### Limiting distribution of a Markov chain

If *P* is the transition probability matrix of an aperiodic, irreducible, finite state Markov chain, then
5

Where ***α*** = [*α*_1_, *α*_2_, …, *α*_*m*_] with 0 < *α*_*j*_ < 1 and . See Bhat ( 1984). The chain with this property is said to be ergodic and has a limiting distribution **π**. The transition probability matrix *P* of such a chain is primitive.

### Recurrence and transience of state

Let *X*_*t*_ be a Markov Chain with state space *S*, then the probability of the first transition to state *j* at the *t*^*th*^ step starting from state *i* is
6

Thus the probability that the chain ever returns to state *j* is


and  is the expected value of first passage time. Further, if *i* = *j*, then;
7

and  is the mean recurrence time of state *i* if state *i* is recurrent.

A state *i* is said to be recurrent (persistent) if and only if, starting from state *i*, eventual return to this state is certain. Thus state *i* is recurrent if and only if
8

A state *i* is said to be transient if and only if, starting from state *i*, there is a positive probability that the process may not eventually return to this state. This means *f*_ii_^*^ < 1

## Model specification

### Defining the problem (Equity price changes as a three-state Markov process)

Let *Y*_*t*_ be the equity price at time *t* where *t* = 0, 1, 2, …, *n* (*t* is measured in weekly time intervals). Further, we define *d*_*t*_ = *Y*_*t*_ - *Y*_*t*-1_ which measures the change in equity price at time *t*. Considering each closing week’s price as discrete time unit for which we define a random variable *X*_*t*_ to indicate the state of equity closing price at time *t*, a vector spanned by 0, 1, 2


Next, we define an indicator vector
9

Then clearly for the outcome of *X*_*t*_ we have
10

where . Hence estimates of the probability that the equity price reduce, did not change and increased can be obtained respectively by
11

For the stochastic process *X*_*t*_ obtained above for *t* = 1, 2, …, *n* we can obtained estimates of the transition probabilities *P*_*ij*_ = Pr (*X*_*t*_ = *j*|*X*_*t*-1_ = *i*) for *j* = 0, 1, 2 by defining


where *k* + 1 is the number of states of the chain.
12a

Therefore, an estimate for the transition matrix for *k* = 2 is
12b

Suppose the data in Additional file 1 is uploaded as .csv, then *R* code for computing estimates in (12b) can be found in Additional file 2 (three-state Markov Chain function column). 

### For a two-state Markov process

We maintain the above defined terms and set


further set *i*, *j* = 0, 1, (for *k* = 1) and apply (9), (10), (11), (12a), and (12b) sequentially, we obtain


without loss of generality, suppose *X*_*t*_ has state space *s* = {0, 1} and transition probability matrix
13

Then, *f*_00_^(1)^ = 1 - *θ* and for *n* ≥ 2, we have;


By the Markov property and the definition of conditional probability, we have
14

solving  to obtain the respective mean recurrence time. Thus,
15

Similarly, we have
16

With the corresponding R algorithm shown in Additional file 2 (two-state Markov Chain function column). 

## Generating eigen vectors for computation of limiting distributions

After the transition probabilities are obtained for both two-state and three-state chains, the R codes in the lower portions of columns one and two in Additional file 2 were used to generate the respective eigen vectors for computation of limiting distributions. 

## Findings and discussions

### Data structure and summary statistics

Data used for this paper are weekly trading price changes for five randomly selected equities on the Ghana Stock Exchange (GSE), each covering period starting from January 2012-December 2013. We obtain the weekly price changes using the relation *d*_*t*_ = *Y*_*t*_ - *Y*_*t*-1_ where *Y*_*t*_ represents the equity closing price on week *t* and *Y*_*t*-1_ is the opening price for the immediate past week. The equities selected include Aluworks (ALW), Cal Bank (CAL), Ecobank Ghana (EBG), Ecobank Transnational Incorporated (ETI), and Fan Milk Ghana Limited (FML).

**Figure 1:**
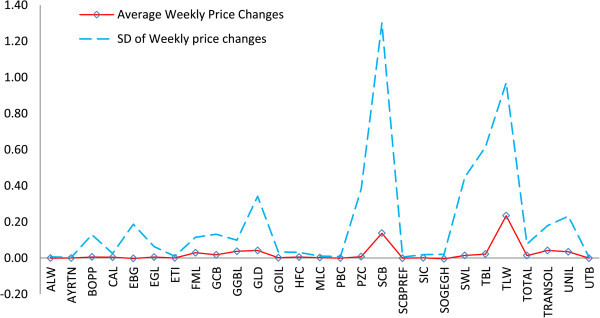
**A plot of mean and standard deviation of weekly price changes of equities.** The plot indicates a very volatile weekly market price fluctuation for any market participating investor. This indicates high level of risk associated with equity purchase decision. We consider that the rational investor would basically seek to maximize purchasing decisions faced with this risk.

In all, 104 (52 weeks) observational data points where obtained. Summary statistics on all respective equities on the GSE are shown in Table 1. We present summaries on the respective number of weekly price decreases, no change in price and price increase. Descriptive statistics for each equity weekly price change is also shown.Overall, the frequency of "no price change" was more experienced over the study period. The lowest and highest price changes for the trading period are respectively -4.19 and 9.54. The estimated values of the kurtosis and skewness are also shown. Figure 1 presents a plot of the average weekly equity price changes of respective equities listed on the GSE over the study period in comparison to the standard deviation of weekly price changes.

**Table 1:** **Summary statistics on the weekly trading price change over the study period**

	Number of weekly price change			Weekly price change summary						
	Decrease	No change	Increase	Mean	SD	Max	Min	Skew.	Kurt.	Count
ALW	15	77	12	0.00	0.01	0.01	-0.04	-2.30	14.23	104
AYRTN	8	89	7	0.00	0.00	0.01	-0.01	-0.10	4.39	104
BOPP	26	45	33	0.01	0.13	0.44	-0.62	-1.80	11.79	104
CAL	27	40	37	0.00	0.03	0.12	-0.07	1.69	7.26	104
EBG	30	44	30	0.00	0.19	0.50	-1.60	-5.65	51.32	104
EGL	21	46	37	0.01	0.06	0.39	-0.25	1.05	15.79	104
ETI	18	59	27	0.00	0.01	0.04	-0.04	-0.71	5.62	104
FML	22	38	44	0.03	0.12	0.85	-0.19	4.08	25.22	104
GCB	25	37	42	0.02	0.13	0.79	-0.41	1.68	12.51	104
GGBL	5	51	48	0.04	0.10	0.73	-0.20	3.84	22.70	104
GLD	7	79	18	0.04	0.34	3.13	-0.72	7.22	64.43	104
GOIL	16	53	35	0.00	0.03	0.12	-0.23	-2.99	23.63	104
HFC	8	75	21	0.01	0.03	0.27	-0.08	5.76	47.80	104
MLC	7	75	22	0.00	0.01	0.05	-0.03	1.04	5.42	104
PBC	13	81	10	0.00	0.01	0.04	-0.02	1.53	11.02	104
PZC	22	55	27	0.01	0.38	3.02	-1.00	4.78	39.15	104
SCB	38	40	26	0.14	1.30	9.54	-4.19	4.45	30.38	104
SCBPREF	11	87	6	0.00	0.01	0.01	-0.03	-2.35	9.67	104
SIC	19	67	18	0.00	0.02	0.16	-0.06	5.55	50.15	104
SOGEGH	12	89	3	0.00	0.02	0.01	-0.18	-6.56	50.60	104
SWL	9	84	11	0.01	0.45	3.15	-2.00	2.28	27.27	104
TBL	21	62	21	0.02	0.62	2.99	-3.00	0.16	12.33	104
TLW	16	56	32	0.23	0.97	6.56	-1.97	3.77	19.38	104
TOTAL	16	66	22	0.01	0.08	0.52	-0.16	4.89	29.16	104
TRANSOL	12	63	29	0.04	0.18	1.26	-0.50	4.24	24.87	104
UNIL	3	76	25	0.03	0.23	1.79	-0.77	5.23	38.66	104
UTB	12	87	5	0.00	0.01	0.02	-0.02	-1.30	6.18	104

### Empirical results on model application (three-state Markov chain)

For the five randomly selected equities, the transition probabilities of the equities are presented as follows. These were obtained from equation (12a) defining  w.r.t. the three-state space Markov process. A 3 × 3 transition matrix is obtained for respective equities as defined by (12b).

From the results of the algorithm, we select 5 equities with which we implement the hypothesis. They include;


Clearly,  for all *i*, *j* = 0, 1, 2 indicating irreducibility of the chains for all equities. Hence state 0 for all the equities is aperiodic and since periodicity is a class property, the chains are aperiodic. These imply that the chains are ergodic and have limiting distributions.

**Figure 2:**
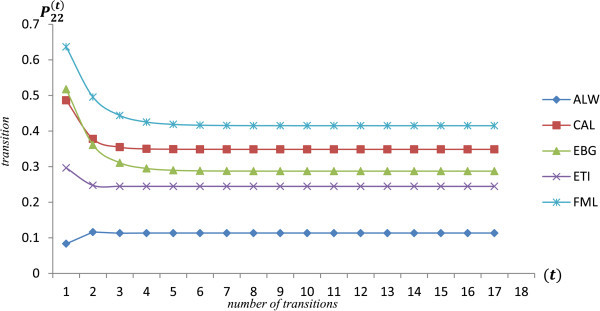
**t-step transition probabilities for share price increases.**

Figure 2 presents the *t* - *step* transition probabilities for share price increases based on the assumption of time-homogeneity. This shows linear plot of transition probabilities for *P*_22_^(*t*)^ for each selected stock as computed above. It measures the probability that a share at initial state (*i. e. state* 2) at inception transited to *state* 2 again after *t weeks*. Regarding the plot of the transition probabilities, the logical reasoning is to choose the equity which has the highest *P*_22_.

From the plot, *FML* share is the best choice for the investor since the probability that it increases from a high price to another higher price is higher when compared to the other selected stocks. ALW recorded the least probability of transition within the period. Comparing CAL to EBG, the methodology shows that CAL shares maintain high probability of moving to higher prices as compared to EBG shares although the later started with high prices at inception.

Using equation (5), the limiting distributions of the respective equities were computed. These probabilities measure the proportions of times the equity states within a particular state in the long run. From Table 2, ALW equity has 14% chance of reducing and 11% chance of increasing in the long run. It however has 75% chance of no change in price. Similarly, in the long run, FML equity has 20% chance of reducing, 39% chance of experiencing no change in price and 42% chance of increasing in price. It is easily seen that for this instance, FML equity has the highest probability of price increase in the long run.

**Table 2:** **Entries of the limiting distribution at for respective equities**

Equity	Limiting distribution		
	***α***_1_	***α***_2_	***α***_3_
**ALW**	0.141509	0.745283	0.113208
**CAL**	0.244980	0.406396	0.348625
**EBG**	0.269568	0.443025	0.287407
**ETI**	0.168470	0.586912	0.244618
**FML**	0.198113	0.386792	0.415094

### Empirical model application (the two-state Markov process)

Defining a two-state space Markov process following from equation (13), we derive the state transition probabilities. The two-state transition probability matrix entries are indicated in Table 3 below;

**Table 3:** **Entries of two-state transition matrices for selected equities**

Equities	***P***_00_	***P***_01_	***P***_10_	***P***_11_
	1 - ***θ***	***θ***	***β***	1 - ***β***
**ALW**	0.133333	0.866667	0.142857	0.857143
**CAL**	0.296296	0.703704	0.227848	0.772152
**EBG**	0.433333	0.566667	0.210526	0.789474
**ETI**	0.166667	0.833333	0.170455	0.829545
**FML**	0.380952	0.619048	0.152941	0.847059

Applying equations (15) and (16) to the transition probabilities, we obtain the respective mean return time of the selected equities. These are shown in Table 4 below;

**Table 4:** **Expected mean return time for respective stocks**

Equity	***μ***_00_	***μ***_11_
		
ALW	1.1555556	7.4285714
CAL	1.3837280	3.6060127
EBG	1.5488889	2.8218623
ETI	1.2009132	5.9772727
FML	1.4497355	3.2235294

**Figure 3:**
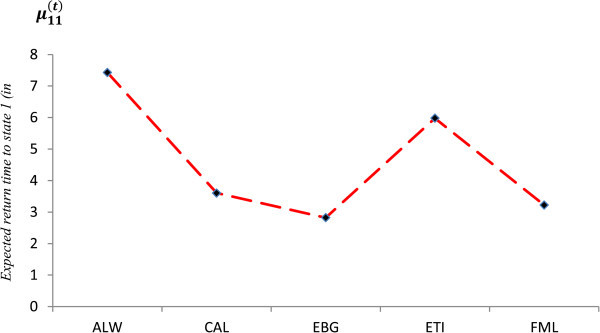
**Mean recurrence time of selected shares.**

Mean return time is measured in weeks with *μ*_*ij*_ as defined in (15) and (16). The mean return time measures the expected time until the equity price’s next return to the state it was initially in at time 0. Figure 3 presents a plot of expected return time of the selected stocks at *μ*_11_. This determines the expected time until the next increase in share. We expect that the choice of share should not only have the highest transition probability, but should relatively possess a lower mean return time. Possessing the least mean return time for *μ*_11_ signifies the shortest return time to a price increase.

## Conclusion

The Markov Process provides a credible approach for successfully analyzing and predicting time series data which reflect Markov dependency. The study finds that all states obtained communicate and are aperiodic and ergodic hence possessing limiting distributions. It is distinctive from Figures 1 and 2 (expected return time and t-step state transition probabilities of equity price increases i.e. *P*_*ij*_ transition from state 2 to state 2) that the investor gains good knowledge about the characteristics of the respective equities hence improving decision making in the light return maximization. With regards to the selected stocks, FML equity recorded the highest state transition probabilities, highest limiting distribution but the second lowest mean return time to price increases (i.e. 3.224 weeks).

Our suggested use of Markov chains as a tool for improving stock trading decisions indeed aids in improving investor knowledge and chances of higher returns given risk minimization through best choice decision. We showed that the proposed method of using Markov chains as a stochastic analysis method in equity price studies truly improves equity portfolio decisions with strong statistical foundation. In our future work, we shall explore the case of specifying an infinite state space for the Markov chains model in stock investment decision making.

## Electronic supplementary material

Additional file 1: **Weekly Price Change Data for GSE.** (DOCX 22 KB)

Additional file 2: **R algorithm for respective methodologies.** (DOCX 14 KB)
