# Supplementary material for: A methodology for stochastic analysis of share prices as Markov chains with finite states
Source: Springerplus. 2014 Nov 6;3:657. doi: 10.1186/2193-1801-3-657 (PMC4247363; doi:10.1186/2193-1801-3-657)
Supplement: Supplementary file 1 — Additional file 1: Weekly Price Change Data for GSE. (DOCX 22 KB) [file 40064_2014_1373_MOESM1_ESM.docx]

**Additional file 1. Weekly Price Change Data for GSE**

| Week | ALW | CAL | EBG | ETI | FML |  | Week | ALW | CAL | EBG | ETI | FML |  | Week | ALW | CAL | EBG | ETI | FML |
| --- | --- | --- | --- | --- | --- | --- | --- | --- | --- | --- | --- | --- | --- | --- | --- | --- | --- | --- | --- |
| 1 | 0.00 | -0.02 | -1.60 | 0.02 | -0.10 |  | **37** | -0.01 | 0.03 | -0.02 | 0.01 | 0.02 |  | **73** | 0.00 | 0.00 | 0.01 | 0.00 | 0.00 |
| 2 | -0.01 | -0.02 | 0.00 | 0.01 | -0.13 |  | **38** | -0.01 | 0.08 | -0.33 | 0.01 | 0.00 |  | **74** | 0.00 | -0.01 | 0.00 | 0.01 | 0.11 |
| 3 | 0.01 | 0.02 | 0.00 | -0.01 | -0.06 |  | **39** | -0.01 | 0.04 | 0.00 | 0.00 | 0.24 |  | **75** | 0.00 | 0.00 | -0.01 | 0.00 | 0.00 |
| 4 | 0.00 | -0.05 | -0.06 | 0.00 | 0.08 |  | **40** | 0.01 | 0.00 | 0.03 | 0.00 | 0.00 |  | **76** | 0.01 | 0.01 | 0.00 | -0.01 | 0.06 |
| 5 | 0.00 | 0.00 | -0.02 | -0.04 | -0.02 |  | **41** | 0.00 | 0.00 | 0.15 | -0.04 | 0.06 |  | **77** | 0.00 | 0.00 | 0.02 | 0.00 | 0.35 |
| 6 | 0.00 | -0.01 | -0.01 | 0.01 | -0.08 |  | **42** | 0.00 | 0.00 | 0.00 | 0.00 | 0.12 |  | **78** | 0.00 | 0.00 | 0.04 | 0.00 | 0.17 |
| 7 | 0.00 | -0.02 | -0.35 | 0.00 | -0.08 |  | **43** | 0.00 | 0.00 | 0.00 | -0.01 | 0.10 |  | **79** | 0.00 | 0.01 | 0.01 | 0.00 | 0.02 |
| 8 | 0.00 | 0.00 | -0.15 | 0.00 | -0.09 |  | **44** | 0.00 | 0.06 | -0.10 | 0.01 | 0.02 |  | **80** | 0.00 | 0.02 | 0.01 | 0.01 | 0.00 |
| 9 | -0.01 | -0.02 | 0.40 | 0.00 | -0.08 |  | **45** | 0.00 | -0.01 | -0.03 | 0.01 | 0.02 |  | **81** | 0.00 | 0.01 | -0.06 | 0.00 | 0.00 |
| 10 | 0.01 | 0.00 | 0.00 | 0.00 | 0.00 |  | **46** | 0.00 | 0.00 | 0.17 | 0.02 | 0.03 |  | **82** | 0.00 | 0.01 | -0.04 | 0.00 | 0.03 |
| 11 | -0.01 | 0.00 | 0.01 | 0.00 | 0.00 |  | **47** | -0.01 | 0.02 | 0.24 | 0.04 | 0.13 |  | **83** | 0.00 | 0.00 | 0.00 | 0.00 | 0.03 |
| 12 | 0.00 | -0.01 | 0.00 | 0.01 | 0.00 |  | **48** | 0.00 | -0.02 | 0.23 | -0.03 | 0.02 |  | **84** | -0.01 | 0.00 | -0.05 | 0.00 | -0.06 |
| 13 | 0.00 | -0.02 | 0.37 | -0.01 | -0.05 |  | **49** | 0.00 | 0.11 | 0.00 | 0.00 | 0.23 |  | **85** | 0.00 | -0.01 | 0.05 | 0.00 | -0.19 |
| 14 | 0.00 | 0.00 | 0.00 | 0.01 | 0.00 |  | **50** | 0.00 | -0.02 | 0.30 | 0.02 | 0.85 |  | **86** | 0.00 | 0.01 | 0.01 | 0.00 | 0.00 |
| 15 | -0.01 | 0.01 | -0.01 | -0.01 | -0.03 |  | **51** | 0.00 | 0.00 | 0.17 | 0.01 | 0.43 |  | **87** | 0.00 | 0.01 | 0.01 | -0.01 | 0.00 |
| 16 | 0.00 | 0.00 | 0.00 | 0.00 | 0.00 |  | **52** | 0.01 | -0.01 | 0.01 | 0.02 | -0.02 |  | **88** | 0.01 | 0.01 | -0.01 | 0.00 | -0.02 |
| 17 | -0.01 | -0.01 | 0.05 | 0.00 | 0.00 |  | **53** | 0.00 | -0.02 | 0.00 | 0.00 | 0.00 |  | **89** | 0.00 | 0.00 | 0.00 | 0.00 | 0.00 |
| 18 | 0.00 | 0.01 | 0.02 | 0.01 | 0.13 |  | **54** | 0.00 | 0.00 | 0.00 | 0.00 | 0.00 |  | **90** | 0.01 | 0.00 | 0.00 | 0.00 | 0.02 |
| 19 | 0.00 | -0.01 | 0.50 | 0.00 | 0.10 |  | **55** | 0.00 | 0.01 | 0.00 | 0.00 | 0.00 |  | **91** | 0.01 | -0.01 | -0.04 | 0.00 | 0.00 |
| 20 | 0.01 | -0.02 | 0.10 | 0.00 | 0.08 |  | **56** | 0.01 | 0.06 | 0.00 | 0.00 | 0.05 |  | **92** | -0.01 | 0.01 | -0.01 | 0.01 | 0.00 |
| 21 | 0.00 | -0.07 | 0.00 | -0.01 | 0.00 |  | **57** | 0.00 | 0.01 | -0.01 | 0.00 | 0.05 |  | **93** | 0.00 | -0.01 | -0.02 | 0.01 | 0.00 |
| 22 | 0.01 | -0.06 | 0.00 | -0.01 | 0.05 |  | **58** | 0.00 | 0.01 | -0.03 | 0.00 | 0.00 |  | **94** | 0.00 | 0.00 | 0.00 | 0.00 | 0.00 |
| 23 | 0.00 | 0.02 | 0.00 | 0.00 | 0.01 |  | **59** | 0.00 | 0.01 | 0.00 | 0.01 | 0.00 |  | **95** | 0.00 | 0.00 | -0.05 | 0.00 | -0.02 |
| 24 | 0.00 | 0.04 | -0.04 | 0.00 | 0.01 |  | **60** | 0.00 | 0.01 | 0.00 | 0.00 | -0.02 |  | **96** | 0.00 | -0.01 | 0.00 | 0.01 | -0.01 |
| 25 | -0.01 | 0.12 | 0.00 | 0.00 | -0.01 |  | **61** | 0.00 | 0.00 | 0.00 | 0.00 | 0.00 |  | **97** | 0.00 | 0.00 | 0.01 | 0.00 | 0.01 |
| 26 | 0.00 | 0.06 | 0.00 | 0.00 | 0.00 |  | **62** | 0.00 | 0.00 | -0.05 | 0.00 | 0.01 |  | **98** | 0.00 | 0.00 | -0.03 | 0.01 | 0.00 |
| 27 | 0.00 | 0.00 | 0.00 | 0.01 | 0.00 |  | **63** | 0.00 | 0.00 | 0.01 | 0.00 | 0.01 |  | **99** | -0.04 | 0.00 | -0.01 | 0.00 | 0.00 |
| 28 | 0.00 | 0.00 | 0.00 | -0.01 | 0.10 |  | **64** | 0.00 | 0.00 | -0.01 | -0.01 | 0.04 |  | **100** | 0.00 | 0.01 | -0.07 | 0.00 | 0.00 |
| 29 | 0.00 | -0.01 | 0.00 | -0.01 | 0.10 |  | **65** | 0.00 | 0.00 | -0.07 | 0.00 | 0.20 |  | **101** | 0.00 | 0.00 | 0.02 | 0.00 | 0.00 |
| 30 | 0.00 | 0.01 | 0.00 | 0.00 | 0.04 |  | **66** | 0.00 | 0.01 | 0.01 | 0.00 | 0.05 |  | **102** | -0.01 | 0.00 | 0.00 | 0.00 | 0.00 |
| 31 | 0.00 | 0.02 | 0.00 | 0.01 | 0.06 |  | **67** | 0.00 | 0.00 | 0.00 | 0.00 | 0.00 |  | **103** | 0.00 | -0.01 | 0.00 | 0.00 | 0.01 |
| 32 | 0.00 | 0.05 | 0.00 | -0.02 | 0.00 |  | **68** | 0.00 | 0.00 | 0.00 | 0.01 | 0.00 |  | **104** | 0.00 | -0.01 | 0.00 | 0.01 | -0.02 |
| 33 | 0.01 | 0.00 | 0.00 | -0.01 | -0.10 |  | **69** | 0.00 | 0.00 | 0.00 | 0.00 | 0.12 |  | **105** | 0.00 | 0.01 | 0.05 | -0.01 | 0.00 |
| 34 | 0.00 | 0.01 | 0.00 | 0.01 | 0.01 |  | **70** | 0.00 | 0.00 | -0.01 | 0.00 | 0.05 |  | **106** | 0.00 | 0.00 | 0.01 | 0.00 | 0.00 |
| 35 | 0.00 | 0.00 | 0.00 | 0.00 | -0.11 |  | **71** | -0.01 | 0.01 | 0.00 | 0.00 | 0.05 |  | **107** | 0.00 | 0.00 | 0.02 | 0.00 | -0.05 |
| 36 | 0.00 | 0.01 | 0.01 | 0.00 | 0.00 |  | **72** | 0.00 | -0.01 | 0.00 | -0.01 | 0.00 |  |  |  |  |  |  |  |
