# Supplementary material for: A methodology for stochastic analysis of share prices as Markov chains with finite states
Source: Springerplus. 2014 Nov 6;3:657. doi: 10.1186/2193-1801-3-657 (PMC4247363; doi:10.1186/2193-1801-3-657)
Supplement: Supplementary file 2 — Additional file 2: R algorithm for respective methodologies. (DOCX 14 KB) [file 40064_2014_1373_MOESM2_ESM.docx]

**Additional file 2 R algorithm for respective methodologies**

| THREE STATES MARKOV CHAIN FUNCTION R<-function(D)  {DIFFDATA=as.numeric(D)  DIFF <- DIFFDATA  for(i in 1:length(DIFF)){  X<-function(DIFF){  if (DIFF<0) X=0  else if (DIFF==0) X=1 else X=2  }  x<-c(lapply(DIFF,X))  }  X<-as.numeric(x)  t=seq(1:length(X))  I<-function(i){  ifelse(X[t] == i,1,0)  }  n<-function(i){sum(I(i),na.rm=T)}  n1=sum(c(n(0),n(1),n(2)))  p<-function(i){n(i)/n1}  t=seq(1:length(X))  N<- function(i,j) {  return(ifelse(X[t]==i&X[t+1]==j,1,0))  }  P<-function(i,j){sum(N(i,j),na.rm=T)/n(i)  }  for(i in 0:2){  for(j in 0:2){  P(i,j)}  TP<-matrix(c(P(0,0),P(1,0),P(2,0),P(0,1),P(1,1),P(2,1),P(0,2),P(1,2),P(2,2)),3,3)  colnames(TP)=c("D","S","I");rownames(TP)=c("D","S","I")  }  i=c(1:3)  j=c(1:3)  s<-function(b){  alpha=0.1  c=1  r=(1-2*min(TP[i,j]))  nthlimit=(log10(alpha)-log10(c))/log10(r)  nthlimit  }  s1<-function(b){  TP1=TP%*%TP  alpha=0.1  c=(1-2*min(TP1[i,j]))^(-1)  r=(1-2*min(TP1[i,j]))^1/2  nthlimit=(log10(alpha)-log10(c))/(log10(r))  nthlimit  }  nth<-function(){  ifelse(all(c(TP[i,j])>0),s(b),(s1(b)+3))  }  A=eigen(t(TP))  A=Re(A$vec[,1])  STP=A/sum(A)  Power <- function(P,n){  A <-diag(rep(1,dim(P)[1]))  for(i in 1:n)  {A <- A %*% P}  return(A)  }  K=Power(TP,nth())  rownames(K)=c("D","S","I")  NUMBSTATES=c(n(0),n(1),n(2))  nthweek=nth()  PROBABILITIES=c(p(0),p(1),p(2))  options(digits=2)  TRANSPROB=TP  STATIONDIST= STP  LIMITPROB=K  return(list(NUMBSTATES=c(n(0),n(1),n(2)),PROBABILITIES=c(p(0),p(1),p(2)),nthweek=nth(),TRANSPROB=TP,STATIONDIST=STP, LIMITPROB=K))  } | TWO STATES MARKOV CHAIN FUNCTION R<-function(D)  {DIFFDATA=as.numeric(D)  DIFF <- DIFFDATA  for(i in 1:length(DIFF)){  X<-function(DIFF){  if (DIFF<0) X=0  else if (DIFF==0) X=1 else X=2  }  x<-c(lapply(DIFF,X))  }  X<-as.numeric(x)  t=seq(1:length(X))  I<-function(i){  ifelse(X[t] == i,1,0)  }  n<-function(i){sum(I(i),na.rm=T)}  n1=sum(c(n(0),n(1),n(2)))  p<-function(i){n(i)/n1}  t=seq(1:length(X))  N<- function(i,j) {  return(ifelse(X[t]==i&X[t+1]==j,1,0))  }  P<-function(i,j){sum(N(i,j),na.rm=T)/n(i)  }  for(i in 0:2){  for(j in 0:2){  P(i,j)}  TP<-matrix(c(P(0,0),P(1,0),P(2,0),P(0,1),P(1,1),P(2,1),P(0,2),P(1,2),P(2,2)),3,3)  colnames(TP)=c("D","S","I");rownames(TP)=c("D","S","I")  }  i=c(1:3)  j=c(1:3)  s<-function(b){  alpha=0.1  c=1  r=(1-2*min(TP[i,j]))  nthlimit=(log10(alpha)-log10(c))/log10(r)  nthlimit  }  s1<-function(b){  TP1=TP%*%TP  alpha=0.1  c=(1-2*min(TP1[i,j]))^(-1)  r=(1-2*min(TP1[i,j]))^1/2  nthlimit=(log10(alpha)-log10(c))/(log10(r))  nthlimit  }  nth<-function(){  ifelse(all(c(TP[i,j])>0),s(b),(s1(b)+3))  }  A=eigen(t(TP))  A=Re(A$vec[,1])  STP=A/sum(A)  Power <- function(P,n){  A <-diag(rep(1,dim(P)[1]))  for(i in 1:n)  {A <- A %*% P}  return(A)  }  K=Power(TP,nth())  rownames(K)=c("D","S","I")  NUMBSTATES=c(n(0),n(1),n(2))  nthweek=nth()  PROBABILITIES=c(p(0),p(1),p(2))  options(digits=2)  TRANSPROB=TP  STATIONDIST= STP  LIMITPROB=K  return(list(NUMBSTATES=c(n(0),n(1),n(2)),PROBABILITIES=c(p(0),p(1),p(2)),nthweek=nth(),TRANSPROB=TP,STATIONDIST=STP, LIMITPROB=K))  } |
| --- | --- |
